# Multisensory Perception of Contradictory Information in an Environment of Varying Reliability: Evidence for Conscious Perception and Optimal Causal Inference

**DOI:** 10.1038/s41598-017-03521-2

**Published:** 2017-06-09

**Authors:** Mohammad-Ali Nikouei Mahani, Saber Sheybani, Karin Maria Bausenhart, Rolf Ulrich, Majid Nili Ahmadabadi

**Affiliations:** 1 Cognitive Systems Lab, School of Electrical and Computer Engineering, College of Engineering, University of Tehran, Tehran, Iran; 2 Cognition and Perception, Department of Psychology, University of Tübingen, Tübingen, Germany; 3 School of Cognitive Science, Institute for Research in Fundamental Sciences (IPM), Tehran, Iran

## Abstract

Two psychophysical experiments examined multisensory integration of visual-auditory (Experiment 1) and visual-tactile-auditory (Experiment 2) signals. Participants judged the location of these multimodal signals relative to a standard presented at the median plane of the body. A cue conflict was induced by presenting the visual signals with a constant spatial discrepancy to the other modalities. Extending previous studies, the reliability of certain modalities (visual in Experiment 1, visual and tactile in Experiment 2) was varied from trial to trial by presenting signals with either strong or weak location information (e.g., a relatively dense or dispersed dot cloud as visual stimulus). We investigated how participants would adapt to the cue conflict from the contradictory information under these varying reliability conditions and whether participants had insight to their performance. During the course of both experiments, participants switched from an integration strategy to a selection strategy in Experiment 1 and to a calibration strategy in Experiment 2. Simulations of various multisensory perception strategies proposed that optimal causal inference in a varying reliability environment not only depends on the amount of multimodal discrepancy, but also on the relative reliability of stimuli across the reliability conditions.

## Introduction

Optimal integration of multisensory information received from different sensory organs is crucial for a coherent perception of the complex environment. Many studies have reported benefits of multisensory compared to unisensory perception in psychophysical tasks. Integration of different modalities when they are congruent and synchronous^1–3^ leads to a significant decrease in response time^4–6^, an increase in reliability, and an increase in accuracy^7–14^. However, when information is incongruent across different sensory modalities, integration may lead to a biased percept. Various models of information processing, including causal inference and calibration, have been suggested to describe perception when different modalities receive inconsistent information^15, 16^.

Multisensory causal inference is the process of deciding which sensory inputs originate from the same cause^17, 18^. It plays a crucial role in multisensory perception, especially when there is a discrepancy among the modalities. Previous studies have reported that the probability of assuming a common cause decreases with the increase of this discrepancy^19^. It has also been shown that causal inference across different modalities not only depends on temporal, spatial, and contextual features of the stimuli^20^ but also on prior knowledge and experiences^21, 22^. According to ideal observer models^19^, causal inference in multisensory perception is often performed in line with Bayes’ rule^16^ and in such a way to minimize the estimation error, defined as the mean squared error between the true value and the estimated value of the perceptual system.

In order to cope with inconsistent sensory information, the brain has to discover whether the discrepancy is due to a random noise in the sensory information, or to a systematic error, potentially in one of the sensory systems^18^. Recent studies have suggested that if the source of the discrepancy is a systematic error, calibration is beneficial to resolve the persisting discrepancy. Meanwhile, the accuracy of the sensory systems is maintained throughout calibration^18, 23^. Calibration models describe how unimodal sensory percepts could be changed so that the inconsistency between unimodal percepts decreases. Classical calibration models indicated that all the sensory modalities are calibrated corresponding to vision, known as visual dominance^24, 25^. Recent studies have proposed general calibration models which involve other modalities as well. In such models, all of the modalities are calibrated in accordance with each other based on the relative reliability^15, 26^ of their corresponding cues, assuring minimum-variance sensory estimates over time^15, 27^. However, Zaidel *et al*.^28^ reported independence of calibration from cue reliabilities in the context of a visual-vestibular setup. They proposed a fixed-ratio calibration model in which each modality contributes to calibration with a certain weight that remains constant irrespective of variations of cue reliability.

Although calibration adjusts perception in the case of systematic errors, it is still not clear under which conditions calibration takes place. It seems that multisensory causal inference and multisensory calibration are tied together, that is, calibration of different modalities according to each other would be reasonable only when signals originate from the same external event. Even though the interaction of causal inference and calibration is still unclear, the minimal error in the integration of multimodal contradictory information can be achieved if causal inference and Bayesian integration processes are considered jointly^19^.

Consequently, several processes underlying multisensory perception of incongruent information have been suggested. In order to better understand the mechanisms of perception on one hand and calibration in a multimodal environment on the other hand, it is important to study interactions across these processes. However, some of these interactions might emerge especially under more realistic conditions rather than in the restricted environment that is usually studied in multisensory perception experiments. In natural environments, the reliability of the information received from each organ is often not stationary but can change within a short period of time. For example, when one drives in foggy (or rainy) weather, the reliability of the visual information can change within a short period when the density of the fog varies. Thus, the reliability of the information is not stationary in natural scenarios. Therefore, when studying the interaction of different perceptual processes such as causal inference and calibration, one should consider the role of varying reliabilities. This might be especially informative, for example, in order to identify the conditions which are required for the calibration process, or to investigate the flexibility of the processes underlying multisensory calibration. The multisensory processes, such as causal inference and calibration, require experiences that are acquired through previous observations^22, 29^. In an environment of varying reliability, the question is raised whether the causal inference problem is solved based on the aggregated information across all reliability conditions, or whether it is solved for each reliability condition separately.

**Figure 1:**
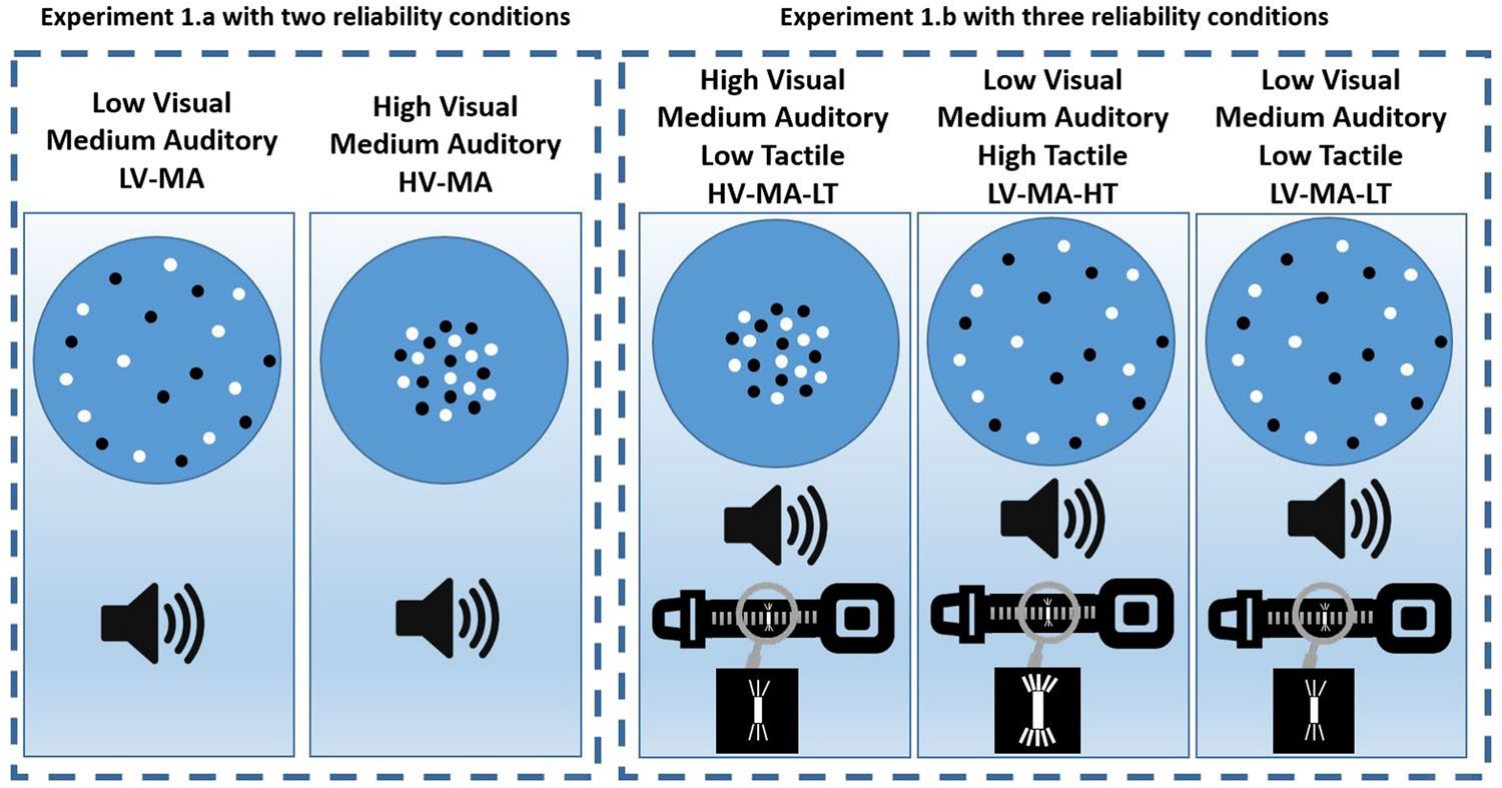
Reliability conditions in Experiment 1.a and Experiment 1.b. Experiment 1.a was a visual-auditory experiment with two reliability conditions (the two left-most figures). The spread of the visual dots was wider in the LV-MA condition than in the HV-MA condition. Experiment 1.b had three reliability conditions (the three right-most figures). In the HV-MA-LT condition, the visual input had the highest reliability, while in the LV-MA-HT condition the tactile stimulus was the most reliable stimulus. In the LV-MA-LT condition, the reliability of both the visual and tactile stimuli was low. The reliability of the auditory stimulus was the same in all the three conditions of Experiment 1.b.

To the best of our knowledge, multisensory calibration and multisensory causal inference of contradictory information have never been studied under conditions where the reliability of the sensory modalities varies randomly from trial to trial within a single session. Therefore, the present study investigates multisensory perception of contradictory spatial information with a concurrent variation of reliabilities. Specifically, we designed an experiment comprised of three sections administered in the following order: a visual-auditory section, a visual-tactile-auditory section, and a unimodal section. In every trial of each section, participants received two consecutive section-specific stimuli presented from varying spatial locations. They were asked to determine whether the second stimulus was perceived spatially on the left or on the right side of the first stimulus (see figures in methods and apparatus section). In the first section, the reliability of the visual stimuli was high in half of the trials, while the reliability of the auditory stimuli was constant in all trials (see Fig. 1). Visual-auditory stimuli had a spatial discrepancy; the auditory stimulus was always presented on the left side of the visual stimulus with a conflict angle (Δ) of −4°. In the visual-tactile-auditory section, tactile stimuli (delivered to the abdomen) accompanied the visual-auditory stimuli. The reliability of the various modalities changed according to three different reliability conditions (see Fig. 1). In this second section, the tactile stimulus was presented 4 cm to the left of the spatially congruent audiovisual stimuli, where the central position of the tactile stimulus was the vertical body axis. The term cue-conflict refers to the spatial conflict between different individual modalities in our study. In this section, cue-conflict means that tactile stimulus was shifted two motors (4 cm) to the left. The third and final section was a unimodal section in which the reliability of each modality was evaluated separately for each participant.

Since the cue-conflict was not large, it was expected that the participants would initially assume a common cause, and hence integrate the different modalities at the beginning of a multimodal section. However, during the course of the experiment, one could expect that participants would realize that there is a systematic conflict rather than unsystematic noise. Accordingly, during each section, the participants’ integration strategy might change. We therefore investigated how their spatial perception varied between the first half and the second half of each section. We hypothesized that three different perceptual strategies could emerge in this setup: (1) Assuming the same cause for the modalities and continuing to integrate the different modalities without any calibration or any change in perception. (2) Assuming the same cause for the modalities, but calibrating the individual modalities in order to resolve the cue-conflict. (3) Inferring that there are several causes for the modalities and thus selecting the most reliable one in each trial.

The first strategy implies that there is no significant change in the perception, specifically between the first and the second half of each section in the experiment. However, the second and the third possibilities represent new strategies of perception. In this study, for the sake of simplicity, we use the term “adaptation” to indicate such possible perceptional changes between the first and the second half of a section, independent of the specific perception strategy. Crucially, we were interested in determining whether these adaptation strategies would depend on the varying reliability of the signals.

As the environment becomes more dynamic, the uncertainty in decisions should increase. Even though it is reported that people are usually aware of their own uncertainty during their perception^30^, there is no consensus on this issue in the literature. Faivre *et al*.^31^ showed that people integrate multimodal information unconsciously when the stimuli were subliminal and after undergoing a conscious learning procedure. Nevertheless, they did not ask their participants to report their confidence about their decisions. Such confidence ratings assess a participant’s awareness of his/her perceptual performance. Specifically, participants demonstrate a high level of monitoring accuracy when their confidence and perceptual performance are positively correlated. Confidence ratings have also been used to assess whether a brain process is conscious or unconscious^32^. For example, if the variation in perceptual performance is not accompanied by consistent changes in confidence, then the perceptual processes and the resulting decisions are likely to be unconscious^33^. In our study, we also assessed the participants’ confidence ratings in order to examine whether the participants were aware of the perceptual difficulty associated with the varying reliabilities of the stimuli.

## Results

### Psychometric function

**Figure 2:**
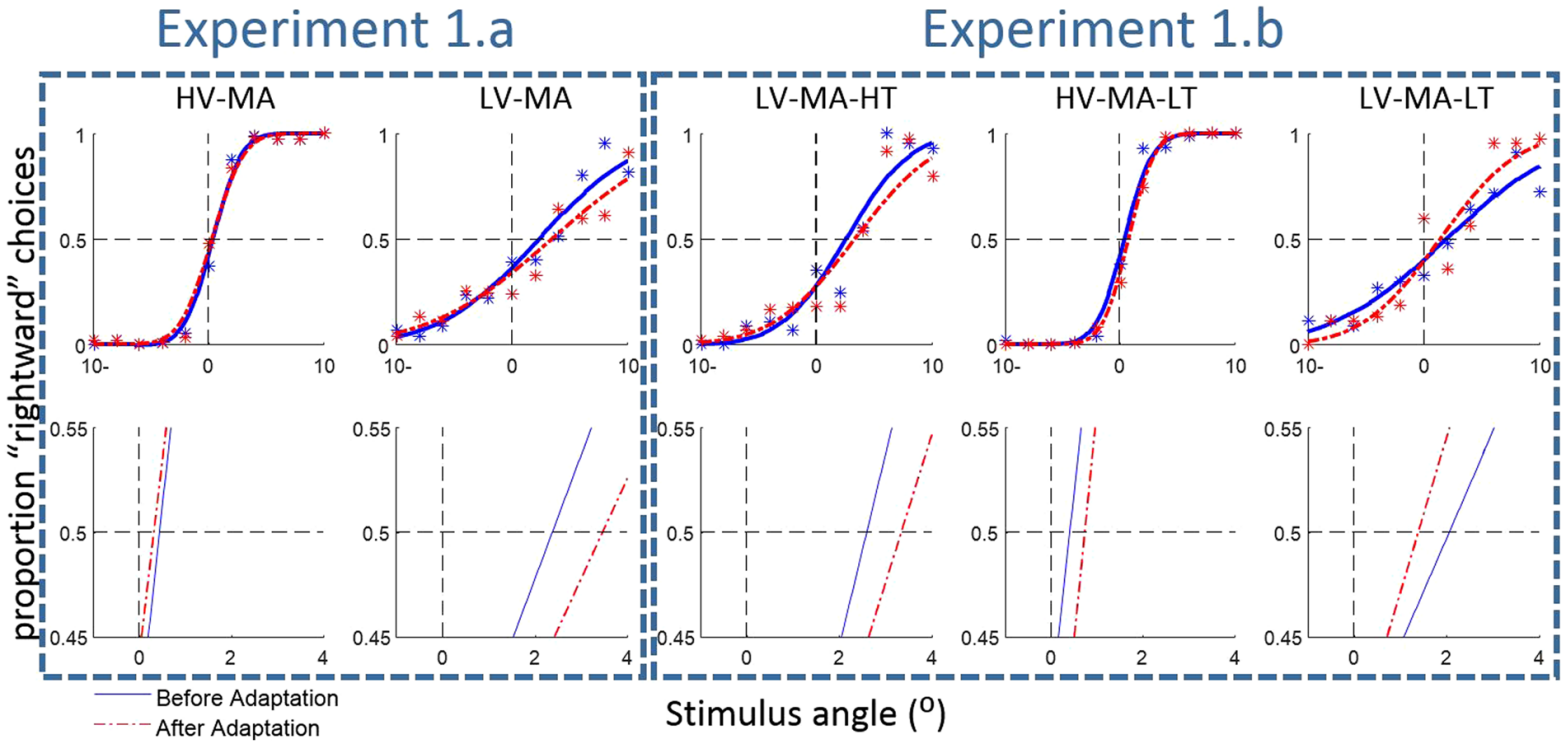
Fitted psychometric functions to all trials of all participants. The first row shows the psychometric functions before and after adaptation in all reliability conditions. The second row shows the magnified plots of the first row around the midpoint.

As outlined above, comparing the point of subjective equality (PSE) for each reliability condition between the first half and the second half of the experiment assesses the strategy adopted by the participant during the experiment. This comparison addresses PSE shifts towards either the visual, auditory, or tactile cues and thus reveals the relative weighting of each modality in the integration process for each reliability condition. The observed psychometric functions measured the proportion of rightward choices against the stimulus angle. Therefore, PSE = −4 means a shift of four degrees to the right and PSE =  +4 a shift of four degrees to the left. We used generalized linear model (GLM) regression in order to fit all psychometric functions. These are maximum likelihood models with the binomial distribution and the probit as the link function (the inverse of the transformation is the link function). The PSE was calculated as the threshold value of the psychometric function at 0.5. Figure 2 illustrates the fitted psychometric function (averaged over participants) for each reliability condition; the solid blue lines show the performance before adaptation and dashed red lines show the performance after adaptation. The mean squared error of these fits is 0.0058 ± 0.0049.

Comparison of the PSEs before and after adaptation (see the magnified plots) illustrates that the PSE changed in some reliability conditions. In a further analysis, psychometric functions were fitted to the data of each participant in each reliability condition. All further analyses and modeling were based on these individual psychometric functions. The average mean squared error of these individual psychometric functions was 0.018 ± 0.016 which indicates a reasonable fit. The average correlation between individual psychometric functions and data points was 0.902 ± 0.085 (average p-values. 014 ± 0.020).

### Visual-Auditory (Experiment 1.a)

In this part of the experiment, the strategy adopted by participants to cope with the visual-auditory perception in an environment of varying reliability was studied. Participants were exposed to two reliability conditions in this section: the high reliability visual and medium reliability auditory stimuli (HV-MA), and the low reliability visual and medium reliability auditory (LV-MA). All trials of the two conditions were randomly mixed. Figure 1 illustrates all reliability conditions in Experiment 1.a and Experiment 1.b.

**Figure 3:**
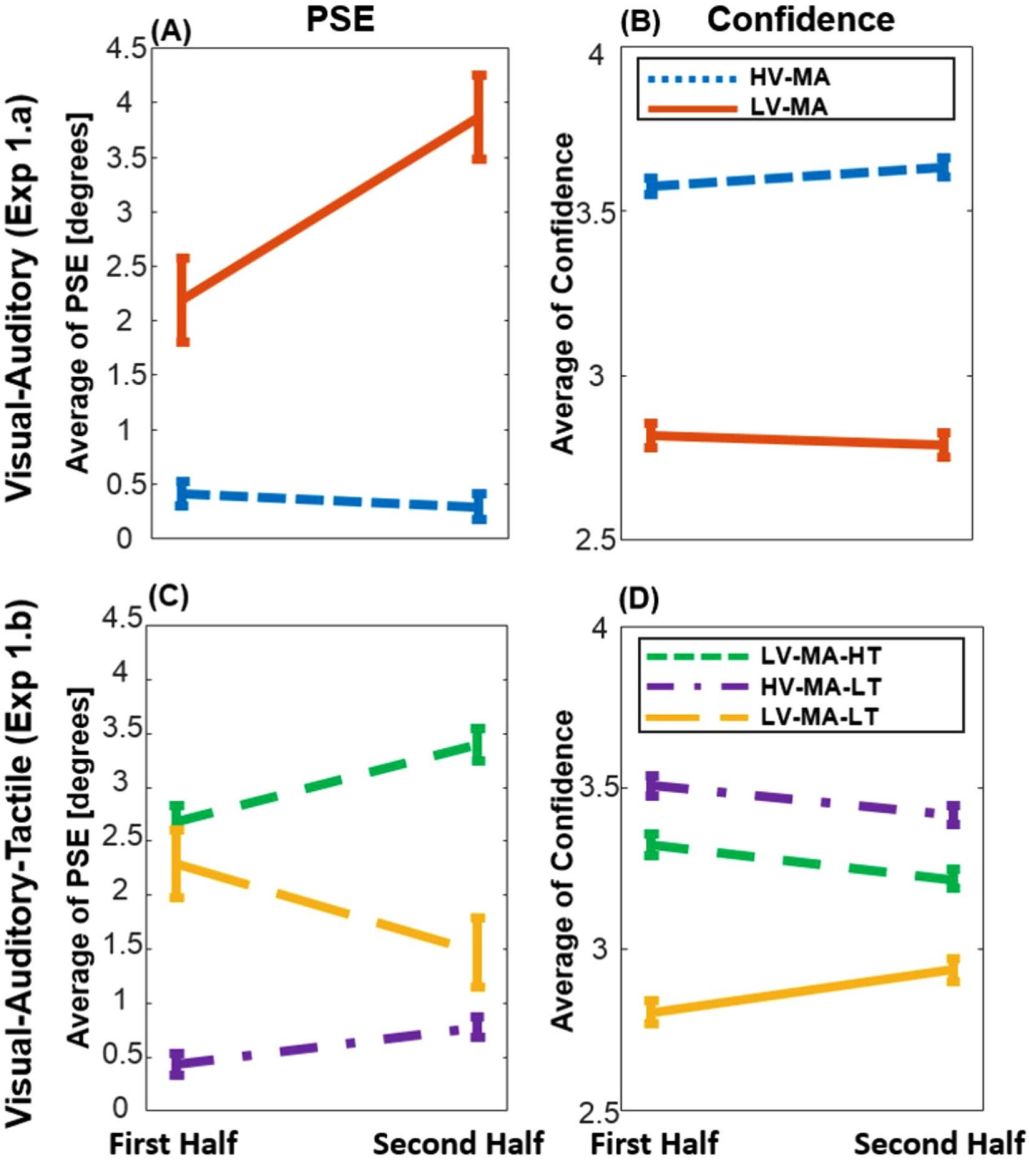
PSE variations as well as confidence changes between the first and the second half of each section across different reliability conditions. The error bars were computed by the method described in ref. 34 and show ± 1SE. (**A**) PSE variation in the visual-auditory section: The PSE was shifted toward the auditory location in the LV-MA condition, while it remained close to the visual location in the HV-MA condition. (**B**) Confidence alteration in the visual-auditory section: there was no significant change in the confidence, however the confidence in the HV-MA condition is significantly higher than in the LV-MA. (**C**) PSE variation in the visual-tactile-auditory section: the PSEs in the HV-MA-LT and the LV-MA-HT conditions moved toward the tactile location significantly. Nevertheless, it was not significant in the LV-MA-LT condition (yellow line). (**D**) Confidence alteration in the visual-tactile-auditory section: All confidence ratings changed significantly, a significant increase in the LV-MA-LT condition, and significant decrease in the HV-MA-LT and the LV-MA-HT conditions.

In order to examine the potential effect of *reliability condition* (LV-MA vs. HV-MA) and of *adaptation* (first half vs. second half) on perception, the PSEs were analyzed using 2 × 2 within-subjects ANOVA (Bayesian analyses are available in the Supplementary material). This analysis showed a significant interaction effect, *F*(1,18) = 5.52, *p* = 0.030, and a significant effect of reliability condition, *F*(1,18) = 72.52, *p* < 0.001; while factor adaptation was not significant, *F*(1,18) = 3.43, *p* = 0.080 (see Fig. 3A). Since the interaction effect was significant, the PSE changes were assessed for each reliability condition. According to a two-sided paired-sample t-test, the effect of adaptation on the PSE was not significant in the HV-MA condition, t(18) = 0.53, *p* = 0.599, but significant in the LV-MA condition, t(18) = 2.17, *p* = 0.043. Therefore, in the second half of the LV-MA condition, participants’ judgments about the multisensory location were significantly biased towards the auditory location. However, the PSE in the HV-MA condition did not change and remained close to the location of the visual stimuli. This pattern of results indicates that perceptual changes are specific to different reliability conditions. We will investigate the source of this change later by modeling these effects.

The slope of psychometric functions was analyzed with the same ANOVA design as the PSE. The results showed that the slope in the HV-MA condition was significantly greater than in the LV-MA condition, *F*(1,18) = 23.59, *p* < 0.001. Neither the factor adaptation, *F*(1,18) = 0.17, *p* = 0.682, nor the interaction of adaptation × reliability condition yielded a significant effect, *F*(1,18) = 0.12, *p* = 0.733. The average slope of the psychometric function was 2.09 ± 2.25 and 1.85 ± 2.14 in the first half and second half of the HV-MA condition, respectively. In the LV-MA condition, the average slope was 0.171 ± 0.08 and 0.148 ± 0.09 in the first half and second half. The difference between the slopes in the HV-MA and LV-MA conditions suggests that our manipulation of the reliability condition was successful.

An analogous ANOVA was performed on confidence ratings. This analysis revealed no significant interaction between reliability condition and adaptation, *F*(1,18) = 1.73, *p* = 0.205. There was also no significant effect of adaptation, *F*(1,18) = 0.14, *p* = 0.712. Nevertheless, the effect of the reliability condition on confidence was highly significant, *F*(1,18) = 112.28, *p* < 0.001, see Fig. 3B. These results showed that participants reported significantly lower confidence when the reliability of stimuli was also low (the LV-MA condition) which supports the view that they were aware of their performance. However, confidence ratings did not significantly differ between the first and second half of the experiment, even when they changed their judgments in LV-MA conditions.

### Visual-Tactile-Auditory (Experiment 1.b)

The second section of the experiment had the same structure as the first section, except that the tactile modality was added and three varying reliability conditions were implemented: a high reliability visual (HV-MA-LT) condition, a high reliability tactile (LV-MA-HT) condition, and a medium reliability auditory (LV-MA-LT) condition (see Fig. 1 and also methods section for more details). Although many studies have mentioned interactions between touch and the other modalities in the context of adaptation^15, 35, 36^, those which have studied the spatial adaptation are rare^37^. Similar to the first section, the difference in the PSEs between the first half and the second half of the experiment is crucial to study the adaptation strategy among the modalities.

The PSE results of this section are shown in Fig. 3C. The 3 × 2 within-subjects ANOVA on PSEs demonstrated that the interaction effect of factor adaptation and reliability (HV-MA-LT, LV-MA-HT, and LV-MA-LT) was significant, *F*(2,36) = 4.53, *p* = 0.018. It also indicated that the effect of adaptation on PSE was not significant, *F*(1,18) = 0.082, *p* = 0.777, while the effect of the reliability condition on PSE was significant, *F*(2,36) = 41.64, *p* < 0.001. The significant interaction effect indicates that adaptation varied across reliability conditions. Therefore, we conducted a separate 2 × 2 within-subjects ANOVA without the LV-MA-LT condition, since the LV-MA-LT condition had a different adaptation trend. This second ANOVA revealed that the interaction between HV-MA-LT and LV-MA-HT was not significant, whereas the effect of reliability condition on PSE, *F*(1,18) = 138.93, *p* < 0.001, and also the effect of adaptation on PSE, *F*(1,18) = 7.23, *p* = 0.015, were significant. The paired-sample t-test also showed that the effect of adaptation on the PSE was not significant in the LV-MA-LT condition, t(18) = 1.30, *p* = 0.209.

The PSE in the LV-MA-HT condition was shifted towards the tactile location, suggesting an alteration of multisensory perception. The same alteration was seen in the condition in which the high reliability stimuli was visual (HV-MA-LT), even though confidence was higher in the LV-MA-HT condition. However, for the LV-MA-LT condition, there was no indication of such variation in the PSE since audition was not affected by touch/vision and the auditory stimuli were the most reliable stimuli in the LV-MA-LT reliability condition (see the slope analyses in the “unimodal study Experiment 1.c section”).

An analogous ANOVA on the slope of the psychometric function revealed that the effect of the reliability condition was significant; *F*(2,36) = 12.08, *p* < 0.001. However, neither factor adaptation, *F*(1,18) = 0.83, *p* = 0.374, nor the interaction of adaptation x reliability condition, *F*(2,36) = 2.39, *p* = 0.106, was significant. Like in Experiment 1.a, the manipulation of the reliability conditions was successful, since the effect of the reliability condition was significant. The slope of psychometric functions in the first half and second half were, respectively, as follows: 0.30 ± 0.15, 0.49 ± 1.08 in the LV-MA-HT condition, 2.08 ± 2.6, 1.14 ± 1.49 in the HV-MA-LT condition, and 0.14 ± 0.10, 0.21 ± 0.09 in the LV-MA-LT condition.

The same ANOVA design was used to study the confidence ratings. When all three modalities are considered together, the 3 × 2 repeated two-way ANOVA on confidence rating showed an significant interaction between reliability and adaptation, *F*(2,36) = 11.47, *p* < 0.001, while factor adaptation was not significant, *F*(1,18) = 0.43, *p* = 0.519, and the effect of reliability condition was significant, *F*(2,36) = 39.05, *p* < 0.001, see Fig. 3D. However, an ANOVA without the LV-MA-LT condition showed that there was no interaction between reliability condition and adaptation, *F*(1,18) = 0.10, *p* = 0.750. Factor adaptation, *F*(1,18) = 6.90, *p* = 0.017, and reliability condition, *F*(1,18) = 7.27, *p* = 0.015, produced significant effects on confidence. Paired-sample t-tests showed that the effect of adaptation on the confidence was also significant in the LV-MA-LT condition, t(18) = 2.49, *p* = 0.023, but in the opposite direction than in the HV-MA-LT and LV-MA-HT conditions. Figure 3D illustrates the changes of the confidence ratings in each reliability condition.

As in experiment 1.a, participants reported significantly different confidence in various reliability conditions suggesting that they were aware of their performance (additional analyses of confidence ratings are provided in the discussion section). In contrast to experiment 1.a, they changed their confidence between the first and the second half of the experiment. Thus, in this section the bias in participants’ perception changed together with their confidence.

### Unimodal study (Experiment 1.c)

In order to model multisensory perception, we need to estimate the unimodal perceptual reliabilities of the participants. Therefore, the last section was a unimodal experiment, designed to estimate the reliability of the stimuli in each of the modalities for each of the participants separately. One-sample t-tests were conducted for each stimulus type in order to examine whether there were any persisting biases in location perception for unimodal stimuli. The average of PSEs, as well as the *p*-values of the t-test, in each of the reliability conditions were as follows: −0.22 ± 0.96, t(18) = 0.99, *p* = 0.335, for high reliability visual, 0.62 ± 3.9, t(18) = 0.69, *p* = 0.498, for low reliability visual, −0.90 ± 1.8, t(17) = 2.08, *p* = 0.052, for high reliability tactile, 3.2 ± 4.6, t(18) = 3.01, *p* = 0.007, for low reliability tactile, and −0.04 ± 1.1, t(18) = 0.15, *p* = 0.880, for medium reliability auditory conditions. One PSE value was eliminated from the analysis in the high reliability tactile condition since it was outside of the average ± 3σ range. Thus, the t-test for this condition was conducted with eighteen data instead of nineteen.

The PSE for low reliability tactile was significantly biased. Specifically, participants displayed a partial bias to the left for the low reliability tactile condition. Since the unimodal experiment was performed after experiment 1.b, this may reflect an acquired shift in location perception corresponding to the previously experienced conflict angle. Presumably, the shift was significant in the low reliability condition because the certainty was lower when the reliability of the stimuli was also lower. Thus, participants probably used their prior information about the conflict angle more when the uncertainty of the stimuli was higher.

A one-way within-subjects ANOVA revealed that there is a significant difference across slopes of the unimodal perceptions, *F*(4,72) = 4.42, *p* = 0.003. The average of the slopes was 0.57 ± 0.25 in the high reliability visual condition, 0.075 ± 0.026 in the low reliability visual condition, 0.58 ± 1.16 in the high reliability tactile condition, 0.12 ± 0.13 in the low reliability tactile condition, and 0.39 ± 0.18 in the medium reliability auditory condition.

The average of the reported confidence was 3.43 ± 0.39 in the high reliability visual condition, 2.72 ± 0.54 in the low reliability visual condition, 3.39 ± 0.37 in the high reliability tactile condition, 2.32 ± 0.75 in the low reliability tactile condition, and 3.02 ± 0.48 in the medium reliability auditory condition. A one-way ANOVA showed that there is a significant difference in confidence across unimodal conditions, *F*(4,72) = 37.74, *p* < 0.001. Post-hoc Tukey test showed that all unimodal confidence ratings were significantly different from each other except two of them: the difference between the high reliability visual and high reliability tactile conditions (*p* = 0.988), as well as the difference between the low reliability tactile and low reliability visual (*p* = 0.110) situations.

In order to account for the results of the experiment, we developed a mathematical model and conducted simulations that explain different strategies by considering the causal inference and calibration processes simultaneously. This model is inspired by the Bayesian framework of multisensory perception and minimizes the overall perception error across all reliability conditions.

## Discussion

### Adaptation strategies in an environment of varying reliability

**Figure 4:**
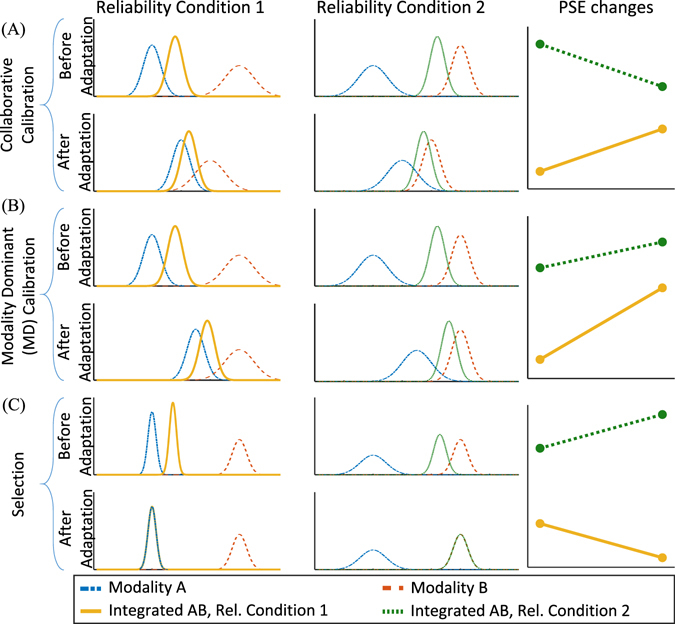
Simulation of different perception strategies in an environment of varying reliability. (**A**) stimulus S is perceived through two sensory modalities A (blue) and B (red) under two different reliability conditions. In the reliability condition 1, the reliability of modality A is higher than the reliability of modality (**B**) while it is vice versa in reliability condition 2. We simulated the variation of integrated PSEs in different adaptation strategies: (**A**) Collaborative calibration, (**B**) Modality Dominant (MD) calibration, and (**C**) Selection.

Our brain faces many perceptual conflicts every day, especially in multimodal perception. Although conflicts seem to be the source of some misperceptions, they often trigger perceptual learning processes. Several studies suggested that cross-modal calibration processes are triggered by cross-modal cue-conflicts^38–40^. The present experiments examined how perception is modified in the presence of cross-modal conflict stimuli in an environment of varying reliability. To enable a better understanding of the results, we simulated different possible multimodal perception strategies in order to model the mechanism that may cause the PSE shifts in an environment of varying reliability (see Fig. 4).

Consider a multimodal stimulus *S* being perceived through two different modalities *A* (blue line), and *B* (red line) and let ${\hat{S}}_{A}={f}_{A}(S)$ and ${\hat{S}}_{B}={f}_{B}(S)$ denote their internal estimations, respectively. Furthermore, several studies^8^ showed that the brain integrates unimodal estimations, ${\hat{S}}_{AB}={w}_{A}\cdot {\hat{S}}_{A}+{w}_{B}\cdot {\hat{S}}_{B}$, in an optimal Bayesian fashion, where the summation of all individual weights (in this case the summation of ${w}_{A}$ and ${w}_{B}$) equals 1. Facing an environment of varying reliability with two reliability conditions, two multisensory stimuli (yellow and green lines) are perceived with the same unimodal but different integrated estimations:

1 $$\{\begin{array}{c}reliability\,condition\,1:{\hat{S}}_{1AB}={w}_{1A}\cdot {\hat{S}}_{A}+{w}_{1B}\cdot {\hat{S}}_{B}\\ reliability\,condition\,2:{\hat{S}}_{2AB}={w}_{2A}\cdot {\hat{S}}_{A}+{w}_{2B}\cdot {\hat{S}}_{B}\end{array}$$

where ${\hat{S}}_{iAB}$ denotes the integrated estimation of stimuli *A* and *B* in the *i* ^*th*^ reliability condition. The difference between the integrated estimations, ${\hat{S}}_{iAB}$ and ${\hat{S}}_{2AB}$, in the two reliability conditions is due to the various reliabilities of unimodal stimuli. However, the unimodal estimations, ${\hat{S}}_{A}$ and ${\hat{S}}_{B}$, do not change across these reliability conditions. The weights are calculated as follow:

2 $${w}_{iA}=\frac{\frac{1}{{\sigma }_{Ai}^{2}}}{\frac{1}{{\sigma }_{Ai}^{2}}+\frac{1}{{\sigma }_{Bi}^{2}}},{w}_{iB}=\frac{\frac{1}{{\sigma }_{Bi}^{2}}}{\frac{1}{{\sigma }_{Ai}^{2}}+\frac{1}{{\sigma }_{Bi}^{2}}}$$

where ${\sigma }_{Ai}^{2}$ and ${\sigma }_{Bi}^{2}$ are the variance of the modality *A* and modality *B* in the *i* ^*th*^ reliability condition.

Comparing the PSE variations in the simulation study and the observed PSE shifts informs us about the strategies taken by participants in the present psychophysical study. Assume that modality *A* receives the more reliable stimulus in reliability condition 1 and modality *B* receives the more reliable stimulus in reliability condition 2, similar to our experimental design. Our simulation shows how the integrated PSEs change dependent upon different adaptation strategies. As discussed before, we assume that three different strategies might occur: Calibration, selection, and no change in the perception. Here, we simulated the selection behavior as well as two modes of calibration. We did not simulate the case when there is no change in the behavior since the PSEs simply would not change. The simulated strategies are as follow: (A) Collaborative calibration: assumes that both of the conflicting modalities shift toward each other in a calibration process. (B) Modality dominant (MD) calibration: one modality is calibrated according to the other modality, so one modality remains steady, called the supervisor modality, and the other modality adapts to the supervisor modality. (C) Selection: the perceptual system selects the most reliable modality, instead of integrating both inputs.

The discrepancy between the modalities is denoted by ${\rm{\Delta }}={\hat{S}}_{A}-{\hat{S}}_{B}$ before the adaptation. In a calibration process the unimodal estimators, *f*_*A*_(*S*) and *f*_*B*_(*S*), are updated in order to decrease the conflict Δ. The collaborative calibration, as well as the MD calibration, were defined as follow:

3 $$Collaborative\{\begin{array}{c}{f}_{A}^{New}={f}_{A}^{Old}-{\rm{\Delta }}{C}_{A}\\ {f}_{B}^{New}={f}_{B}^{Old}+{\rm{\Delta }}{C}_{B}\end{array},\,MD\{\begin{array}{c}{f}_{A}^{New}={f}_{A}^{Old}-{\rm{\Delta }}{C}_{A}\\ {f}_{B}^{New}={f}_{B}^{Old}\end{array}$$

where ${f}_{j}^{Old}$ is the old estimator, ${f}_{j}^{New}$ is the updated estimator of the *j* ^*th*^ modality, and *C*_*j*_ is the calibration coefficient of the *j* ^*th*^ modality. In our simulations, *C*_*A*_ and *C*_*B*_ are equal and positive values in the collaborative calibration while *C*_*B*_ is zero in the MD calibration. After the adaptation, the multisensory stimuli were calculated according to Equation (3) in the collaborative calibration and MD calibration models.

By comparing the simulation and the experimental result (see Figs 3 and 4), it becomes clear that calibration is not the sole strategy the brain takes to overcome a conflict, at least in a varying reliability multisensory environment. Experiment 1.a suggests that when the participants received conflicting visual-auditory stimuli in an environment of varying reliability, they started to select between the visual and the auditory stimulus (Fig. 3A), exhibiting the selection strategy (Fig. 4C). In this situation, the PSEs corresponding to the multimodal stimuli moved toward the location of the unimodal stimuli during the experiment, each one toward the most reliable unimodal stimulus. The integrated PSE of the LV-MA condition (red line) moved toward the auditory source. However, the integrated PSE of the HV-MA condition (blue line) reflected the visual source. This pattern is consistent with the view that participants selected the auditory stimulus in the low reliability visual (LV-MA), and the visual stimulus in the high reliability visual (HV-MA) condition. Undoubtedly, this trend in the changes of the PSEs is inconsistent with each of the two calibration processes and is best described by a selection strategy.

The results of Experiment 1.b correspond to the Modality Dominant calibration strategy (Fig. 4B), suggesting that vision was calibrated by touch which is also consistent with previous findings^35, 41^. According to the results (Fig. 3C), the PSEs in both the HV-MA-LT (purple line) condition and the LV-MA-HT (green line) condition moved toward the tactile source, meaning that touch affected both reliability conditions, probably by calibrating vision.

Furthermore, the three models were quantitatively compared to experimental results. To this end, we minimized the sum of the absolute error between empirical and predicted PSEs using the genetic algorithm^42^. For the data of Experiment 1.a, this sum was 0.01 for the selection model, 0.12 for the MD calibration model, and 1.55 for the collaborative calibration model. Therefore, the results of the Experiment 1.a are best described by the selection model. Likewise, for Experiment 1.b this sum was 0.34 for the selection model, 0.18 for the MD calibration model, and 1.06 for the collaborative calibration model. Accordingly, MD calibration was the dominant strategy in Experiment 1.b.

### Merging the integration model and the selection model

Our experimental results together with the simulation results provide evidence that participants rely on both selection and calibration strategies when facing conflict stimuli in an environment of varying reliability. We have fitted a perceptual model to experimental data that enables us to assess the probability of different perception strategies in experiment 1.a and experiment 1.b.

According to previously suggested models^19^, causal inference in multisensory perception is performed in line with the model averaging approach^16, 17^. In the model averaging approach, the optimal estimate is a weighted average of two estimates: one derived under the assumption that two stimuli originate from different sources, the other derived under the assumption that two stimuli originate from the same source.

We extended the model averaging approach in order to compare the probability of the common cause between experiment 1.a and experiment 1.b. For the sake of simplicity, the prior probability of all unimodal stimuli is assumed to be uniform in our study. According to the model averaging approach, the perception in an environment of varying reliability with two reliability conditions would be as follows:

4 $$\{\begin{array}{c}reliability\,condition\,1:{\hat{S}}_{1}={P}_{C}\cdot {\hat{S}}_{1AB}+(1-{P}_{C})\cdot {\hat{S}}_{A}\\ reliability\,condition\,2:{\hat{S}}_{2}={P}_{C}\cdot {\hat{S}}_{2AB}+(1-{P}_{C})\cdot {\hat{S}}_{B}\end{array}$$

where *P*_*C*_ denotes the probability of the common cause, ${\hat{S}}_{i}$ is the averaged estimation in the *i* ^th^ reliability condition, and ${\hat{S}}_{1AB}$ and ${\hat{S}}_{2AB}$ are Bayesian integrated stimuli which are calculated according to Equation (1). ${\hat{S}}_{A}$/${\hat{S}}_{B}$ is assumed to be the most reliable stimulus in reliability condition 1/2. The ideal observer integrates ${\hat{S}}_{A}$ and ${\hat{S}}_{B}$ with the probability *P*_*C*_, and selects the most reliable stimulus between ${\hat{S}}_{A}$ and ${\hat{S}}_{B}$ with the probability (1−*P*_*C*_).

We have fitted the model of averaging to the average performance of participants. In the experiment 1.a, we assumed *P*_*C*1_/*P*_*C*2_ (probability of common cause in the first/second half of the experiment), *S*_*V*1_/*S*_*V*2_ (location of visual stimulus in the first/second half of the experiment), and *S*_A1_/*S*_A2_ (location of auditory stimulus in the first/second half of the experiment) are free parameters. In the experiment 1.b, ${S}_{T1}$/${S}_{T2}$ (location of tactile stimulus in the first/second half of the experiment) were also added to the list of free parameters. The slope of the unimodal perceptions (which were measured in experiment 1.c) was used as the reliability of the individual modalities in the averaging model. We have minimized the sum of the square of the difference between the estimated and observed PSEs using the genetic algorithm (GA)^42^. The values of *P*_*C*1_ and *P*_*C*2_ were limited between 0 and 1 in the minimization procedure. We imposed the experimental perceptual reliabilities on the model and let the model fit the other parameters to the experimental data. Therefore, comparison between *P*_*C*1_ and *P*_*C*2_ shows how the model predicts the change of strategy. Furthermore, comparison of location of the individual modalities between the first and the second half would reveal the effect of calibration on the perception.

Table 1 illustrates the fitted parameters in experiment 1.a and 1.b where the fitting error were minimum. The model of the experiment 1.a shows a decrease in the *P*_*C*_. It also illustrates that the perception of the auditory stimulus was shifted to the auditory location while the perception of the visual stimulus was almost unchanged. The fitted parameters in experiment 1.a are in favor of changing the strategy from integration to selection, specifically since *P*_*C*_ decreased from first half to second half. In contrast, the *P*_*C*_ was almost constant in experiment 1.b while location of the individual modalities varied between the first half and second half. This pattern is more consistent with the calibration strategy rather than selection strategy. The model proposed that the visual and tactile modalities were shifted toward the tactile location while the auditory modality was shifted toward the location of the visual and auditory stimuli. The possible account for auditory shift is that the biased perception of the auditory stimulus in experiment 1.a affected the perception of the auditory in experiment 1.b. Thus, the auditory stimulus was shifted back to the unbiased location in experiment 1.b.

**Table 1:** Fitted parameters in experiments 1.a and 1.b.

	P_C*1*_	P_C*2*_	S_V*1*_	S_V*2*_	S_A*1*_	S_A*2*_	S_T*1*_	S_T*2*_	Fitting error
Experiment 1.a	0.56	0.18	−0.18	0.00	2.41	3.97	NA	NA	<0.0000
Experiment 1.b	0.49	0.47	−0.20	0.44	2.39	1.23	2.85	3.99	<0.0000

The parameters of the fitted models confirm the findings which were provided by simulation to some extent. However, the modeling reveals that the probability of the integration was about fifty percent at the beginning of experiments 1.a and 1.b. Thus, participants did not integrate the modalities perfectly. This imperfect integration may explain why the reliability of the multisensory perceptions were not always greater than the reliability of the unimodal perceptions as is expected in the Bayesian integration model. The fitted model predicted that the location of the auditory stimulus was shifted in experiment 1.a. Therefore, participants adjusted their auditory perception while they altered their strategy. It seems that the perception of the auditory stimulus was affected by the discrepancy at the beginning of the experiment when the probability of a common cause of the auditory stimulus and visual stimulus was higher. However, the participants seem to have adjusted their perception of the auditory stimulus later when they decided not to integrate the information.

Even though the results show that participants used the selection strategy in experiment 1.a and the calibration strategy in experiment 1.b, other perceptual processes seemed to be involved in both experiments. Therefore, further investigation is required to rule out the effect of other processes and to confirm our findings.

### Model averaging explains the optimality in conflict solving strategy

We have investigated the multisensory perception of contradictory information in an environment of varying reliability and showed that participants use different strategies when facing different situations. However, the rationale for using different strategies in experiments 1.a and 1.b was not yet clear. Therefore, we investigated why it might be optimal to use different strategies in these two experiments. To this end, an ideal observer was modeled that minimized the perceptual estimation error in an environment of varying reliability based on the model averaging approach (equation 4). Since all reliability conditions were mixed and presented randomly, the ideal observer should infer the causality in such a way to minimize the overall error across all reliability conditions. Therefore, the overall estimation error, $Erro{r}_{E}$, for the two reliability conditions is defined as follows:

5 $$Erro{r}_{E}={[{P}_{C}(S-{\hat{S}}_{1AB})+(1-{P}_{C})(S-{\hat{S}}_{A})]}^{2}+{[{P}_{C}(S-{\hat{S}}_{2AB})+(1-{P}_{C})(S-{\hat{S}}_{B})]}^{2}$$

Assuming that *P*_*C*_ is not changing in each trial, an optimal estimation of the common cause probability, *P*_*C*_, is the one which minimizes the *Error*_*E*_ (see the supplementary material for the proof and derivation):

6 $${P}_{C}=\frac{{\hat{S}}_{A}+{\hat{S}}_{B}-2S}{{\rm{\Delta }}(1-{w}_{1A}-{w}_{2A})}$$

According to Equation (6), increasing the discrepancy,Δ, leads to decrease in ${P}_{C}$; that is, the larger the discrepancy, the more probable that selection strategy is chosen by an ideal observer, which is consistent with previous studies investigating multisensory perception^19^. Furthermore, minimizing the error function in an environment of varying reliability reveals a term we call discrepancy weight,

7 $${w}_{{\rm{\Delta }}}=(1-{w}_{1A}-{w}_{2A}),$$

This discrepancy weight shows the gain effect of the discrepancy (weight of the effect of the discrepancy) on *P*_*C*_. Since *w*_*iA*_ = 1*w*_*iB*_, the discrepancy weight, *w*_Δ_, depends on the relative reliability of both stimuli in both reliability conditions. Consequently, the ideal observer chooses between the selection or integration strategies not only according to the amount of the discrepancy Δ, but also based on the relative reliability of the stimuli in all reliability conditions, represented by discrepancy weight *w*_Δ_.

Similar to Equations (5) and (6), the optimal *P*_*C*_ and the discrepancy weight for three modalities and three reliability conditions, as in experiment 1.b, was derived as follows (see the supplementary material for error function, derivation and the proof).

8 $${P}_{C}=\frac{{\hat{S}}_{A}+{\hat{S}}_{B}+{\hat{S}}_{C}-3S}{{\rm{\Delta }}(2-{w}_{1A}-{w}_{2A}-{w}_{3A}-{w}_{1B}-{w}_{2B}-{w}_{3B})}$$

9 $${w}_{{\rm{\Delta }}}=(2-{w}_{1A}-{w}_{2A}-{w}_{3A}-{w}_{1B}-{w}_{2B}-{w}_{3B})$$

Equation (9) indicates the gain effect of the discrepancy on ${P}_{C}$ for three modalities across three reliability conditions.

### Discrepancy weights explain the dissimilarity in perception strategies

**Figure 5:**
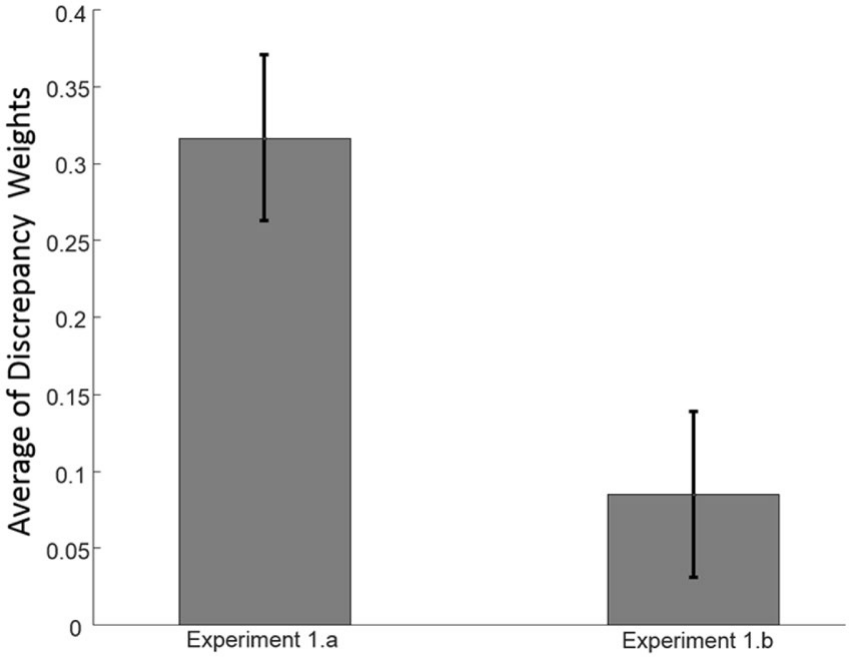
Discrepancy Weights. The discrepancy weights depend on the relative reliability of the cues across the different reliability conditions. Discrepancy weights are inversely correlated to the probability of the common cause *P*_*C*_. Thus, the lower discrepancy weight in the experiment 1.b indicates a higher probability of an assumed common cause, which is consistent with the observed behavior in that experiment. In contrast, the higher discrepancy weight in the experiment 1.a points to a lower common cause probability that explains the selection strategy in experiment 1.a.

Since the amount of the discrepancy (Δ) was the same in experiment 1.a and experiment 1.b, we hypothesize that the discrepancy weight is the source of taking different multimodal perception strategies. To investigate this, we compared the ${w}_{\Delta }$ in experiment 1.a and in experiment 1.b using Equation (7) and Equation (9) respectively. The values of **σ** in Equation (2) are obtained by fitting psychometric functions to the results of the unimodal experiment (experiment 1.c). Figure 5 illustrates the average of the discrepancy weights over all participants in experiments 1.a and 1.b. The paired-sample t-tests showed a significant difference, *t*(17) = 2.14, *p* = 0.047, suggesting that the dissimilarity in taking different strategies is related to discrepancy weight (excluding one outlier whose weights outside of the range average ± 3σ). The lower discrepancy weights in experiment 1.b points to the higher probability of the common cause *P*_*C*_ in that experiment. Therefore, the participants preferred to integrate the information in the visual-tactile-auditory experiment and tended to solve the conflict by calibration. In contrast, the discrepancy weight is higher in the visual-auditory experiment (experiment 1.a), and therefore the probability of the common cause is lower than in the visual-tactile-auditory experiment. As a result, the participants preferred to use the selection strategy to decrease the overall error in the visual-auditory experiment.

### Evidence for conscious adaptation in multisensory environment

**Figure 6:**
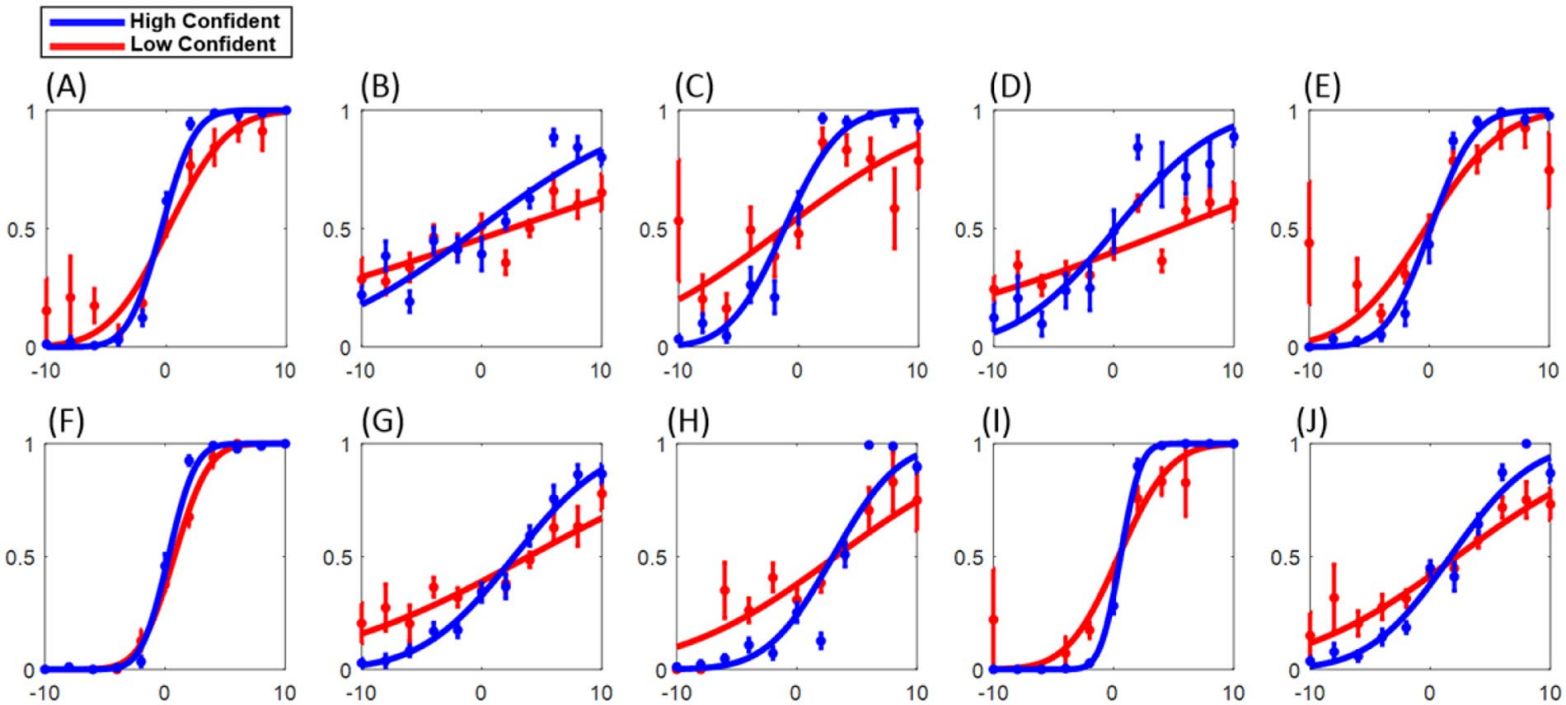
The Performance of overall subjects in high confidence and low confidence groups: (**A**)- Unimodal, high reliability visual. (**B**)- Unimodal, low reliability visual. (**C**)- Unimodal, high reliability tactile. (**D**)- Unimodal, low reliability tactile. (**E**)- Unimodal, medium reliability auditory. (**F**)- Multimodal high reliability visual-medium reliability auditory. (**G**)- Multimodal, low reliability visual-medium reliability auditory. (**H**)- Multimodal, high reliability tactile-low reliability visual-medium reliability auditory. (**I**)- Multimodal, low reliability tactile-high reliability visual-medium reliability auditory. (**J**)- Multimodal, low reliability tactile-low reliability visual-medium reliability auditory. The error bars show the standard deviation for running the bootstrap with 1000 iterations.

Confidence reporting is a metacognitive process which represents the cognition about one’s own judgments^32^. In multisensory perception, it illustrates how much an individual is aware of his/her perceptual performance when coming across a multisensory stimulus. The confidence ratings together with slopes of the psychometric functions provide a measure of the participants’ awareness of their performance^43–45^. In order to investigate the degree of correspondence between the self-reported confidence and the perceptual performance, we partitioned the confidence ratings into high confidence and low confidence trials. The high confidence trials include all trials in which the reported confidence level was equal to or greater than 3 and the low confidence trials consist of the trials with confidence level equal to or less than 2. For multisensory sessions (experiments 1.a and 1.b), a 2 × 5 (high/low confidence level × five reliability conditions) within-subjects ANOVA showed that there is a main effect of confidence level on the slope of the fitted psychometric functions, *F*(1,18) = 7.89, *p* = 0.012, as well as a significant effect of reliability condition, *F*(4,72) = 13.63, *p* < 0.001. However, no significant interaction effect was observed, *F*(4,72) = 1.93, *p* = 0.115. The same result was obtained for the unimodal experiment (experiment 1.c). The 2 × 5 within-subjects ANOVA again showed a main effect of confidence level on the slope, *F*(1,18) = 12.35, *p* = 0.002, as well as a significant effect of reliability condition on the slope, *F*(4,72) = 8.45, *p* < 0.001, but no significant interaction effect, *F*(4,72) = 1.38, *p* = 0.249. The psychometric functions for each experiment were plotted separately for high and low confidence trials (see Fig. 6). If participants were aware of their discrimination performance, better performance (a steeper slope) should be observed in high confidence trials than in low confidence trials. This expectation was clearly confirmed by the above analysis. The corresponding variation between the perceptual performance and the confidence reporting is seen for both unimodal (Fig. 6A–E) and multimodal judgments (Fig. 6F–J).

Furthermore, the variation of the confidence and the PSE in experiment 1.b (Fig. 3C,D) favor conscious calibration; that is, the changes of PSEs regarding the calibration in experiment 1.b were accompanied by a corresponding variation in confidence, thus providing evidence for conscious calibration. However, there was no significant variation in the confidence of experiment 1.a, in which participants used the selection strategy.

### Conclusion

The brain employs multiple perceptual processes such as integration of multimodal stimuli, causal inference, and calibration in order to achieve a coherent perception of the complex and dynamic environment. Even though many studies have investigated and modeled these perceptual processes in multisensory environments, multisensory perception has never been studied when the reliability of the sensory modalities varies within a single session. We investigated the multisensory perception of contradictory information in such an environment of varying reliability. Particpiants were tested in a visual-auditory experiment, where they were asked to discriminate the direction of conflicting visual-auditory stimuli with two different reliability conditions. They also performed the same task in a visual-tactile-auditory experiment with three different reliability conditions.

Our results demonstrate that participants initially started to integrate the information, but later they changed their perception strategy in order to overcome the conflict. They chose the selection strategy in the visual-auditory experiment and the calibration strategy in the visual-tactile-auditory experiment. To understand the rationale for using different strategies, we modeled the perceptual mechanisms in an environment of varying reliability and also provided an ideal observer model. Our model suggests that causal inference in an environment of varying reliability depends not only on the amount of discrepancy but also on the reliability of stimuli across all reliability conditions. Thus, the rationale for using different strategies is probably mediated by the difference in weights of stimuli across reliability conditions. We also investigated the participants’ awareness during the experiment by analyzing their confidence in their judgments. Their performance was better in high confidence trials than in the low confidence trials, indicating that participants were aware of their performance levels during the experiment. Moreover, participants did not change their confidence in Experiment 1.a, when they switched from integration behavior to the selection behavior. However, they changed their confidence in Experiment 1.b in which they calibrated their modalities.

In conclusion, the present study demonstrates that humans employ various strategies in a multimodal environment of varying reliability to cope with inconsistent information. Modeling of the results obtained suggests that it is optimal to utilize different strategies based on the amount of inconsistent sensory information and relative cue reliability. The results also indicate that humans engage consciously in these various perceptual strategies in such an environment.

## Methods and Apparatus

### Participants

20 healthy male subjects (21.61 ± 0.63 years old) participated in the experiment. The data of one participant was excluded from data analysis because his PSE was outside average ± 3σ. None of the participants reported having any history of neurological disorders. They all reported normal or corrected to normal vision, normal hearing, normal tactile sense, and no neurological problems.

All procedures and experimental protocols are approved by the ethical committee board of the University of Tehran, Tehran, Iran. All methods were carried out in accordance with the approved guidelines. A written informed consent was also obtained from all participants prior to data collection and they were compensated 10000 Rials per hour (approx. 3 USD) for their participation.

### Stimuli and Materials

Participants were seated in an armchair in a sound-attenuated room in front of a 19′′ LCD screen with 60 Hz refresh rate, on which the visual stimuli were presented. The distance between participants’ eyes and the screen was 50 cm, corresponding to approximately 40° visual angles.

**Figure 7:**
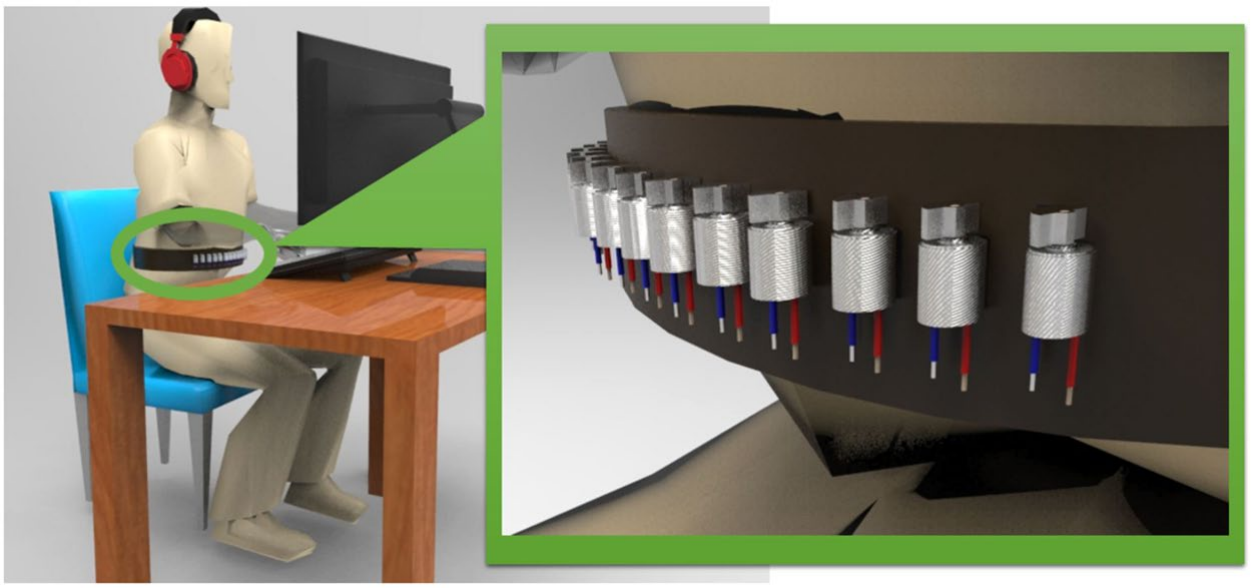
Experimental setup. Participants received tactile stimuli through a vibrotactile belt around their abdomen. The belt consists of 14 minatory vibration motors, which are fixed horizontally on the belt. Running a vibration motor in a specific location on the belt causes a tactile stimulus to a subject. They simultaneously received auditory stimuli through a headphone and visual stimuli through a monitor.

Auditory stimuli were delivered to the participant through headphones (Sennheiser HD419). Tactile stimuli were delivered by a custom designed belt, which was tightened over the participant’s abdomen under the bottom of the sternum. The belt, which is called vibrotactile belt, was designed in the Cognitive Robotics Lab at University of Tehran, see Fig. 7.

### Visual stimuli

The visual stimulus was a random dot cloud made up of ten white and ten black dots against a grey background (#7d7d7d). The diameter of each dot was approximately 0.4 degrees. The average position of all dots typifies the position of the visual stimulus and the spread of the dots corresponds to the reliability of the visual stimulus. The more wide-spread the dots, the lower the reliability of the stimulus. A standard deviation of 0.1 of the total screen size (4 degrees of the visual angle) was used to produce the high reliability visual stimuli while a standard deviation of 0.4 of the screen size (16 degrees of the visual angle) was used for the low reliability stimuli.

### Auditory stimuli

The auditory stimulus was a white noise sound played through stereo headphones. The Head Related Transform Function (HRTF) simulated the position of the origin of the sound. The auditory stimulus was generated by SLAB which is an open source real-time virtual acoustic environment rendering system developed by the Spatial Auditory Displays Lab at NASA Ames Research Center. The intensity of the auditory stimulus was approximately 70 db (SPL). The reliability of the auditory stimuli did not change in the experiment.

### Tactile stimuli

The vibrotactile belt consists of 14 vibration motors which are fixed horizontally on a cotton canvas tape. Running a vibration motor in a specific location on the belt causes a tactile stimulus to the subject. The reliability of the tactile stimuli can be adjusted by a separated PWM (Pulse Width Modulation) level for each vibration motor. The vibration motors are controlled by a microcontroller with a 32 MHz clock. According to a pilot test, the values 100%, and 60% were used as the PWM levels to produce high reliable and low reliable vibrations.

### Experimental Procedure

The experiment included two multisensory and one unimodal spatial perception tasks which consisted of delivering visual, auditory and tactile stimuli to the participants. Each of these sections consisted of trials which only differed in the reliability of the information provided to the stimulated modalities. For example, in one trial, a high reliability visual stimulus may have accompanied a medium reliability auditory stimulus, while in the next trial a low reliability visual stimulus may have accompanied a medium reliability auditory stimulus. This setup, therefore, provided an opportunity to investigate the mechanisms underlying multisensory integration under more realistic conditions than previous setups since it provides an environment of varying reliability.

**Figure 8:**
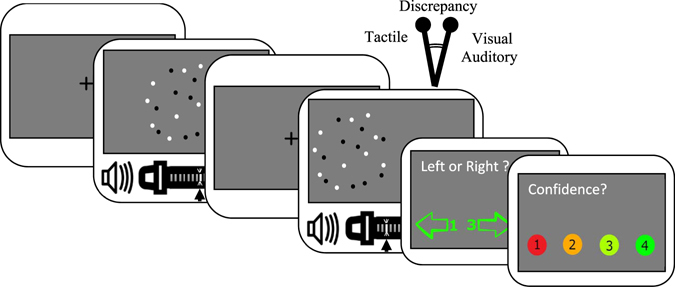
Time course of an exemplary trial: Subjects were presented a fixation screen for 800 ms, and then the first multimodal stimulus for 100 ms. Afterwards, another fixation screen was presented for 600 ms, and the second multimodal stimulus with a cue-conflict (spatial discrepancy of −4° visual angle) was presented for 100 ms. Subjects were asked to report whether the second stimulus was to the left or right of the first one. They also reported their confidence on a scale of 1 (low confidence) to 4 (high confidence).

In multimodal trials, the stimuli from the different modalities were presented simultaneously. In each trial, first a fixation cross was presented for 800 ms. Afterward, two consecutive stimuli were presented, each for 100 ms, separated by an interval of 600 ms, in which again a fixation cross was presented (Fig. 8). After the presentation of the stimuli, the subject was asked if the second stimulus (i.e., the comparison stimulus) was perceived on the left or the right of the first stimulus (i.e., the standard stimulus). The standard stimulus was always presented at the central position, and the location of the comparison stimulus varied between 11 spatial points evenly distributed around fixation along the horizontal axis. In multisensory trials, the judgment should be based on a combined impression from all stimulated senses. After judging the relative position, the participant was asked to report their confidence about the judgment, on a scale of 1 (low confidence) to 4 (high confidence), by pressing the corresponding number on the numeric keypad. The inter-trial interval was 800 ms for all trials.

### Experiment 1.a

Experiment 1a was a visual-auditory spatial perception session. In each trial of this section, the first visual stimulus was always presented at the fixation cross. The second visual stimulus could be presented at 11 different stimulus locations distributed along the horizontal axis on the screen (the distance between fixation and the most left/rightwards presented stimulus was 10° visual angle), with a higher density at the middle of the range, where spatial discrimination should be harder. Each visual stimulus was accompanied by an auditory stimulus. There was no cue conflict between vision and audition for the first stimulus pair (standard stimulus). For the second stimulus pair (comparison stimulus), the auditory stimulus was always presented at a cue-conflict of −4 ° angle from the visual stimulus. There were two sets of trials with two different reliability conditions: In the high-reliability set, visual stimuli were presented at a high reliability and auditory stimuli at a medium reliability (**HV-MA**), and in the low-reliability set, visual stimuli were presented with low reliability and auditory with medium reliability (**LV-MA**). Inside each set, there were 115 trials, covering a symmetric range of 11 spatial points for spatial locations. These points were adjusted to be denser in the middle of the range, where differentiating between the first and the second stimulus in a pair becomes more challenging (15 trials for 5 midpoints). In order to avoid any bias resulting from the order of the trials, both trial sets were randomly mixed.

### Experiment 1.b

The second section was a visual-tactile-auditory spatial perception session, which was completed right after the first section with no break. As in the first section, it consisted of several sets of trials, with varying reliability of the visual and/or tactile stimulation. Auditory stimuli were always presented with medium reliability. For the standard stimulus, the visual, tactile, and auditory stimuli were presented concurrently at the central position. For the comparison stimulus, auditory stimuli were presented at the same spatial position as the visual stimuli, while tactile stimuli were presented at a conflict angle of −4° degrees visual angle from the audiovisual ones. Specifically, three different stimulus sets were presented: **LV-MA-HT:** visual (low reliability), auditory (medium reliability), tactile (high reliability). **HV-MA-LT:** visual (high reliability), auditory (medium reliability), and tactile (low reliability). **LV-MA-LT:** visual (low reliability), auditory (medium reliability), tactile (low reliability). Similar to the first section, there were 115 trials in each trial set, covering a symmetric range of 11 spatial locations. All three trial sets were randomly mixed. After the second section, the subject had a break for about 5 minutes.

### Experiment 1.c

The third section consisted of 575 unimodal trials, comprising five sets of 115 trials each. The stimuli specification in each set, as well as the order of the presentation, was as follows: **1** ^**st**^ **set:** auditory (medium reliability), **2** ^**nd**^ **set:** tactile (high reliability), **3** ^**rd**^ **set:** tactile (low reliability), **4** ^**th**^ **set:** visual (high reliability), **5** ^**th**^ **set:** visual (low reliability). The 2^nd^ and the 3^rd^ set were presented together and in random order, just as the 4^th^ and the 5^th^ set.

### Practice trials

Before the experiment, all participants performed a number of practice trials with instructions provided by an experimenter. The practice section consisted of 50 trials, but it was terminated as soon as the participant felt familiarized with the task. The practice section included only visual-auditory trials.

## Electronic supplementary material

Supplementary Information
