# Supplementary material for: Multisensory Perception of Contradictory Information in an Environment of Varying Reliability: Evidence for Conscious Perception and Optimal Causal Inference
Source: Sci Rep. 2017 Jun 9;7:3167. doi: 10.1038/s41598-017-03521-2 (PMC5466670; doi:10.1038/s41598-017-03521-2)
Supplement: Supplementary file 1 — Supplementary Information [file 41598_2017_3521_MOESM1_ESM.pdf]

Supplementary Material

Multisensory Perception of Contradictory Information in an Environment of Varying Reliability: Evidence for Conscious Perception and Optimal Causal Inference

Mohammad-Ali Nikouei Mahani[1,2,3*], Saber Sheybani[1], Karin Maria Bausenhart[2], Rolf Ulrich[2], Majid Nili Ahmadabadi[1,3]

1- Cognitive Systems Lab, School of Electrical and Computer Engineering, College of Engineering, University of Tehran, Tehran, Iran.

2- Cognition and Perception, Department of Psychology, University of Tübingen, Tübingen, Germany.

3- School of Cognitive Science, Institute for Research in Fundamental Sciences (IPM), Tehran, Iran.

*Corresponding Author: nikouei@ut.ac.ir

Derivation of equations

Here, we show how Equations (6) and (8) in the main article can be derived from minimizing the error function. Assume that the perception is carried out based on the averaging model. Thus, the error function for two reliability conditions is defined as sum of the squared error as follows:

$$Error_E = \left[P_C\left(S - \hat{S}_{1AB}\right) + (1-P_C)\left(S - \hat{S}_A\right)\right]^2 + \left[P_C\left(S - \hat{S}_{2AB}\right) + (1-P_C)\left(S - \hat{S}_B\right)\right]^2 \qquad (1)$$

Assume that the dynamics of P_C, the probability of the common cause, is much slower than S and it needs many observations in order to be updated. Thus, we assumed that P_C is constant and solved the equation $\frac{dError_E}{dS} = 0$ in order to find the optimum S and P_C as follows:

$$\frac{dError_E}{dS} = 2P_C(\mathrm{S} - \hat{S}_{1AB}) + 2P_C(\mathrm{S} - \hat{S}_{2AB}) - 2(\mathrm{S} - \hat{S}_A)(P_C - 1) - 2(\mathrm{S} - \hat{S}_B)(P_C - 1)$$

$$= 2P_C(\mathrm{S} - \hat{S}_A w_{1A} + \hat{S}_B(w_{1A} - 1)) + 2P_C(\mathrm{S}\text{-}\hat{S}_A w_{2A} + \hat{S}_B(w_{2A} - 1)) - 2(\mathrm{S} - \hat{S}_A)(P_C - 1) - 2(\mathrm{S} - \hat{S}_B)(P_C - 1)$$

$$\frac{dError_E}{dS} = 0 \Rightarrow \begin{pmatrix} P_C \\ S \end{pmatrix} \begin{cases} \left\{ \begin{pmatrix} P_C = X \\ S = \frac{\hat{S}_A}{2} + \frac{\hat{S}_B}{2} \end{pmatrix} \middle| X \in (0,1) \right\} \quad if \quad w_{1A} + w_{2A} = 1 \\ \left\{ \begin{pmatrix} P_C = \frac{\hat{S}_A + \hat{S}_B - 2S}{\Delta\left(1 - w_{1A} - w_{2A}\right)} \\ S = Z \end{pmatrix} \middle| Z \in ComplexNumbers \right\} \end{cases}$$

(2)

We extended the aforementioned equations to the situation when there are three modalities and three reliability conditions under the same assumptions as before. Thus, the error in estimation is defined as follows:

$$Error_E = \left[P_C\left(S - \hat{S}_{1ABC}\right) + (1-P_C)\left(S - \hat{S}_A\right)\right]^2 + \left[P_C\left(S - \hat{S}_{2ABC}\right) + (1-P_C)\left(S - \hat{S}_B\right)\right]^2 + \left[P_C\left(S - \hat{S}_{3ABC}\right) + (1-P_C)\left(S - \hat{S}_C\right)\right]^2$$

(3)

Where: $\hat{S}_{iABC} = w_{iA}\hat{S}_A + w_{iB}\hat{S}_B + w_{iC}\hat{S}_C$ (4)

Since the w_{iA}, w_{iB}, w_{iC} are calculated according to the Bayesian model (the extension of Equation (2) in the main text for three modalities), we can rewrite the S_{iABC} as follows:

$$w_{iA} + w_{iB} + w_{iC} = 1 \Rightarrow \hat{S}_{iABC} = w_{iA}\hat{S}_A + w_{iB}\hat{S}_B + (1 - w_{iA} - w_{iB})\hat{S}_C \quad (5)$$

Similar to the two reliability conditions, we solved the equation $\frac{dError_E}{dS} = 0$ to obtain the optimum S and P_C :

$$\frac{dError_E}{dS} = 2P_C(\mathrm{S} - \hat{S}_{1ABC}) + 2P_C(\mathrm{S} - \hat{S}_{2ABC}) + 2P_C(\mathrm{S} - \hat{S}_{3ABC}) - 2(\mathrm{S} - \hat{S}_A)(P_C - 1)$$
$$- 2(\mathrm{S} - \hat{S}_B)(P_C - 1) - 2(\mathrm{S} - \hat{S}_C)(P_C - 1)$$

$$\frac{dError_E}{dS} = 2P_C(\mathrm{S} + \hat{S}_C(w_{1A} + w_{1B} - 1) - \hat{S}_A w_{1A} - \hat{S}_B w_{1B}) + 2P_C(\mathrm{S} + \hat{S}_C(w_{2A} + w_{2B} - 1) - \hat{S}_A w_{2A} - \hat{S}_B w_{2B})$$
$$+ 2P_C(\mathrm{S} + \hat{S}_C(w_{3A} + w_{3B} - 1) - \hat{S}_A w_{3A} - \hat{S}_B w_{3B}) - 2(\mathrm{S} - \hat{S}_A)(P_C - 1) - 2(\mathrm{S} - \hat{S}_B)(P_C - 1) - 2(\mathrm{S} - \hat{S}_C)(P_C - 1)$$

$$\frac{dError_E}{dS} = 0 \Rightarrow$$
$$\left(\begin{array}{c} P_C = \dfrac{\hat{S}_A + \hat{S}_B + \hat{S}_C - 3S}{(\hat{S}_A + \hat{S}_B - 2\hat{S}_C - \hat{S}_A w_{1A} - \hat{S}_B w_{1B} + \hat{S}_C w_{1A} + \hat{S}_C w_{1B} - \hat{S}_A w_{2A} - \hat{S}_B w_{2B} + \hat{S}_C w_{2A} + \hat{S}_C w_{2B} - \hat{S}_A w_{3A} - \hat{S}_B w_{3B} + \hat{S}_C w_{3A} + \hat{S}_C w_{3B})} \\ S = Z \end{array} \right)$$
$$\Rightarrow P_C = \frac{\hat{S}_A + \hat{S}_B + \hat{S}_C - 3S}{\hat{S}_A(1 - w_{1A} - w_{2A} - w_{3A}) + \hat{S}_B(1 - w_{1B} - w_{2B} - w_{3B}) - \hat{S}_C(2 - w_{1A} - w_{1B} - w_{2A} - w_{2B} - w_{3A} - w_{3B})}$$

Similar to Experiment 1.b, we assume that $\hat{S}_A = \hat{S}_B \neq \hat{S}_C$ since the visual and auditory stimuli originate from the same location but differed from the tactile location. Thus, we can simplify the P_C as follows:

$$P_C = \frac{\hat{S}_A + \hat{S}_B + \hat{S}_C - 3S}{\Delta\left(2 - w_{1A} - w_{2A} - w_{3A} - w_{1B} - w_{2B} - w_{3B}\right)}$$

Where $\Delta = \hat{S}_A - \hat{S}_C = \hat{S}_B - \hat{S}_C$.

Bayesian analyses

All of the Bayesian Analyses were done by JASP [1]. JASP files and other codes can be downloaded from https://github.com/manmahani/MP_VaryingRelEnv page.

Bayesian Analyses of PSEs in Experiment 1.a

Bayesian ANOVA

Model Comparison

Models	P(M)	P(M|data)	BF_M	BF_{10}	error %
Null model (incl. subject)	0.200	1.305e -8	5.222e -8	1.000	
Adaptation	0.200	9.250e -9	3.700e -8	0.709	1.047
Reliability Condition	0.200	0.124	0.569	9.535e +6	1.364
Adaptation + Reliability Condition	0.200	0.193	0.958	1.480e +7	2.652
Adaptation + Reliability Condition + Adaptation ✻ Reliability Condition	0.200	0.682	8.593	5.227e +7	2.778

Note. All models include subject.

Analysis of Effects

Effects	P(incl)	P(incl|data)	$BF_{Inclusion}$
Adaptation	0.600	0.876	4.689
Reliability Condition	0.600	1.000	2.989e +7
Adaptation ✻ Reliability Condition	0.200	0.682	8.593

Bayesian T-Test

Bayesian Paired Samples T-Test

			BF_{10}	error %
HV-MA_Before	-	HV-MA_After	0.270	1.591e -4
LV-MA_Before	-	LV-MA_After	1.589	4.113e -5

Inferential Plots

HV-MA_Before - HV-MA_After

Prior and Posterior

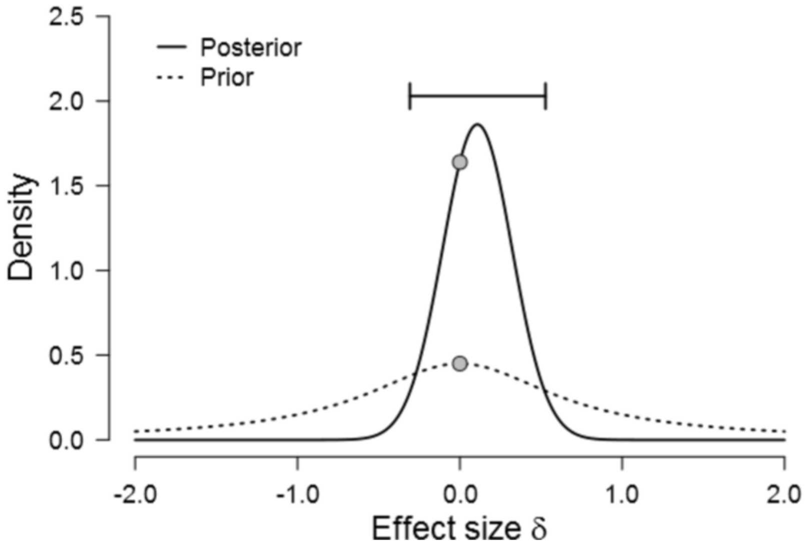

LV-MA_Before - LV-MA_After

Prior and Posterior

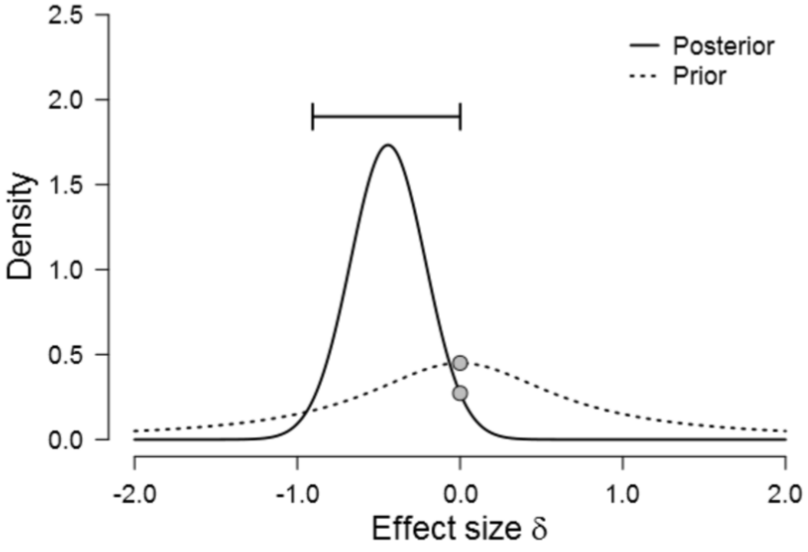

1. Marsman, M. & Wagenmakers, E.-J. Bayesian benefits with JASP. *Eur. J. Dev. Psychol.* 1–11 (2016). doi:10.1080/17405629.2016.1259614
